# Extending the straight leg raise test for improved clinical evaluation of sciatica: reliability of hip internal rotation or ankle dorsiflexion

**DOI:** 10.1186/s12891-021-04159-y

**Published:** 2021-03-24

**Authors:** Janne Pesonen, Michael Shacklock, Pekka Rantanen, Jussi Mäki, Lauri Karttunen, Markku Kankaanpää, Olavi Airaksinen, Marinko Rade

**Affiliations:** 1 Department of Rehabilitation, Kuopio University Hospital, PL100, 70029 KYS, Kuopio, Finland; 2 Department of Surgery (incl. Physiatry), University of Eastern Finland, Kuopio, Finland; 3 Neurodynamic Solutions, Adelaide, Australia; 4 Department of Physical and Rehabilitation Medicine, Helsinki University Hospital, Helsinki, Finland; 5 Department of Physical and Rehabilitation Medicine, Kanta-Häme Central Hospital, Hämeenlinna, Finland; 6 Department of Physical and Rehabilitation Medicine, Tampere University Hospital, Tampere, Finland; 7 Josip Juraj Strossmayer University of Osijek, Faculty of Medicine, Orthopaedic and Rehabilitation Hospital “Prim. dr. Martin Horvat”, Rovinj, Croatia; 8 Department of Natural and Health Studies, Juraj Dobrila University of Pula, Pula, Croatia

**Keywords:** Straight leg raise, Sciatica, Lumbar intervertebral disc herniation, Interrater reliability, Structural differentiation

## Abstract

**Background:**

The straight leg raise (SLR) is the most commonly applied physical tests on patients with sciatica, but the sensitivity and specificity ratings for disc hernia and neural compression leave areas for improvement. Hip internal rotation tensions the lumbosacral nerve roots and ankle dorsiflexion tensions the sciatic nerve along its course. We added these movements to the SLR (extended SLR = ESLR) as structural differentiators and tested inter-rater reliability in patients with LBP, with and without sciatica.

**Methods:**

Forty subjects were recruited to the study by the study controller (SC), 20 in the sciatic group and in the control group. Two independent examiners (E1&E2) performed the ESLR and did not communicate to the subjects other than needed to determine the outcome of the ESLR. First, SLR was performed traditionally until first responses were evoked. At this hip flexion angle, a location-specific structural differentiation was performed to confirm whether the emerged responses were of neural origin. Cohen’s Kappa score (CK) for interrater reliability was calculated for ESLR result in detection of sciatic patients. Also, the examiners’ ESLR results were compared to the traditional SLR results.

**Results:**

The interrater agreement between Examiner 1 and Examiner 2 for the ESLR was 0.85 (*p* < 0.001, 95%CI: 0.71–0.99) translating to almost perfect agreement as measured by Cohen’s Kappa When the ESLR was compared to the traditional SLR, the overall agreement rate was 75% (30/40). Kappa values between the traditional SLR and the E1’s or E2’s ESLR results were 0.50 (*p* < 0.0001; 95%CI 0.27–0.73) and 0.54 (p < 0.0001; 95%CI 0.30–0.77), respectively.

**Conclusions:**

ESLR with the addition of location-specific structural differentiation is a reliable and repeatable tool in discerning neural symptoms from musculoskeletal in patients with radiating low back pain. We recommend adding these movements to the standard SLR with aim of improving diagnostic ability.

## Background

Low back pain (LBP) is a common musculoskeletal ailment worldwide in which radiating leg pain is present in approximately 60% of the patients [1]. Referred pain into the lower extremity is often called sciatica, since it follows the course of the sciatic nerve. Even though there are many possible causes of the radiating pain of sciatica, a commonly considered aspect is mechanical compression of the nerve roots that form the sciatic nerve due to lumbar intervertebral disc herniation [2]. However, with the current knowledge, the causes behind sciatica are known to be much more complex than plain mechanical compression due to lumbar disc herniation, and may vary from inflammatory processes to, for example, neural adhesions, arachnoiditis or virus-induced mononeuritis [2–4]. For instance, a key factor in the SLR is its propensity to reproduce the patient’s pain through sensitivity issues: neuritis can increase mechanosensitivity [5, 6]; the nerve can conduct normally through inflammation when there is no axonal damage [7, 8]; and radiculitis can produce these correlates without pressure on the nerve root [9]. This could explain imperfection of the SLR in diagnosis of nerve root compression.

The straight leg raise (SLR) test is the most commonly performed physical test for diagnosis of sciatica and lumbar disc hernia [10]. The SLR is considered positive when it evokes radiating pain along the course of the sciatic nerve and below the knee between 30 and 70 degrees of hip flexion [2]. Studies of its capacity to diagnose lumbar disc hernia show high sensitivity but heterogeneous/low specificity [10, 11]. The reference standard has usually been magnetic resonance imaging (MRI) and occasionally electrodiagnosis, in which imperfect diagnostic capability may link to heterogeneity in the interpretation of the test.

Bragard test is a modification of the SLR, where ankle dorsiflexion is applied at the end of the SLR. Dorsiflexion reduces the SLR angle at which the test is positive [12] and can be used to discern neural symptoms from musculoskeletal [2]. However, problems with this exist: there is no clear procedural definition and it is unclear whether it applies above 70 degrees. In addition, research on Bragard test is sparse. There is some evidence it increases specificity for detection of sciatic symptoms [13], but its reliability and repeatability have not been studied.

To understand the value (application and effectiveness) of the SLR, it is imperative to acknowledge problems with the reference standards. The prevalence of asymptomatic disc hernias on MRI is high [14], radiologically detected nerve root compression does not always coincide with a ‘positive’ SLR nor clinical symptoms [15, 16] and electrodiagnostic tests do not always detect nerve root lesions [17]. Herein also lies the issue in the literature: The reference standard against which the tests are compared may be imperfect which could render interpretation of the SLR erroneous.

The SLR moves the sciatic nerve up to the nerve roots and a positive test may arise from problems anywhere along this course – thigh, buttock, and spine [18, 19]. Published data on neural movement during the SLR (with or without pathology) [16, 20–24] and the knowledge on all the possible causes of sciatic symptoms imply that SLR may not be at its best when utilized to distinguish solely the presence of LIDH, or other mechanical compression to the nervous structures, but as a test to assess neural mechanosensitivity - without determining a cause. Hence, we modified the SLR to address these known issues.

As low specificity of SLR may be linked to heterogeneity in its interpretation, we addressed the above problems by defining the application and interpretation of an extended SLR (ESLR) for it to detect sciatic patients. We tested ESLR’s interrater reliability to ascertain if hip internal rotation and ankle dorsiflexion would produce consistent responses in patients with LBP, with and without sciatica.

## Methods

The institutional ethics committee granted ethical approval for this study. Subjects were given information about the study and they gave written consent to participate and were able to withdraw from the study at any time. The protocol for this study was designed in accordance with recommendations for reproducibility studies for diagnostic procedures [25] and followed the Declaration of Helsinki.

### Setting and study population

The prevalence of the index condition (P_index_) is synonymous with the frequency of all positive judged diagnostic procedures (the index condition) by the observers. Following this, forty subjects were recruited to the study, 20 to each sciatica and control groups. We recruited subjects to the study of consecutive patients as they appeared in the institutional spine center. The Study Controller initially examined all patients and recruited them after performing a complete clinical examination with a thorough patient history. This was done to determine which patients were likely to have exhibited sciatica and a lumbar nerve root disorder affecting the possible mechanosensitivity and/or mechanical behavior of the lumbosacral nerve roots. The sciatic symptoms were not required to reach below the knee. The subjects allocated to sciatic group were selected using a combination on patient history and clinical findings to detect sciatic patients, and more specifically, with the combination of symptoms/findings of unilateral leg pain, leg pain being worse than back pain, clinical neurological deficits (muscle strength and/or skin sensation, reflexes) and positive neural tension test signs, including SLR and ESLR [26, 27]. The subjects included in the control group reported pain in one or more regions of the low back, greater trochanter and/or hip with or without tightness in the posterior thigh. Complete inclusion and exclusion criteria are shown in Table 1.

**Table 1:**
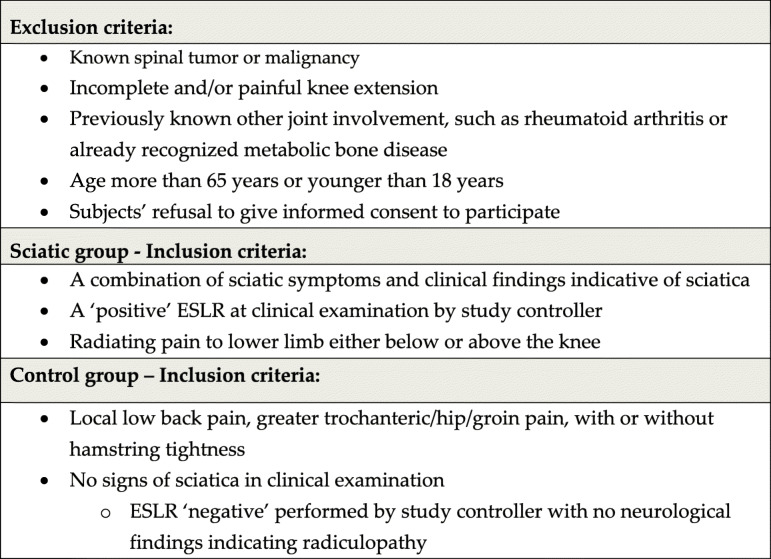
Exclusion and inclusion criteria

ESLR = Extended straigth leg raise test

Two independent examiners (physiatry residents; Examiner 1 and Examiner 2) - blinded from each other’s results - performed the ESLR on the subjects and did not communicate with them other than absolutely necessary to determine the possible reproduction/provocation of the symptoms during the procedure. The data for the traditional SLR result, performed by the treating physician, was extracted from the patients’ medical records and compared with the ESLR results.

### Extended straight leg raise procedure

The ESLR procedure started the similar way as the traditional SLR. The subjects lay supine and with their head in neutral position supported by a standard pillow. The examiner was positioned facing the patient on the same side of the bed as the lifted limb. The examiner’s hands were positioned proximally immediately above patella and distally behind the calf/Achilles tendon. With this grip, the subject’s leg was lifted passively towards 90°with the hip in neutral rotation, knee fully extended and ankle left free, continuing until the first symptoms emerged or symptoms at rest were increased by 30%. In case no responses were evoked, the SLR was ceased at 90°. The patient was informed by the Study Controller to report emerging responses both vocally and by pointing out the area to Examiner. With the sciatic group, ESLR was performed only on the symptomatic side, while in the control group the Study Controller selected the tested side randomly. At the hip flexion angle of evoked responses, a structural differentiation movement (hip internal rotation or ankle dorsiflexion) based on the location of the evoked responses (proximal = buttock/hamstring, or distal = below the knee) was performed to determine whether the symptoms were of neural or musculoskeletal origin. These location-specific maneuvers emphasize nerve movement in the relevant area without moving the adjacent musculoskeletal structures.

**Fig. 1:**
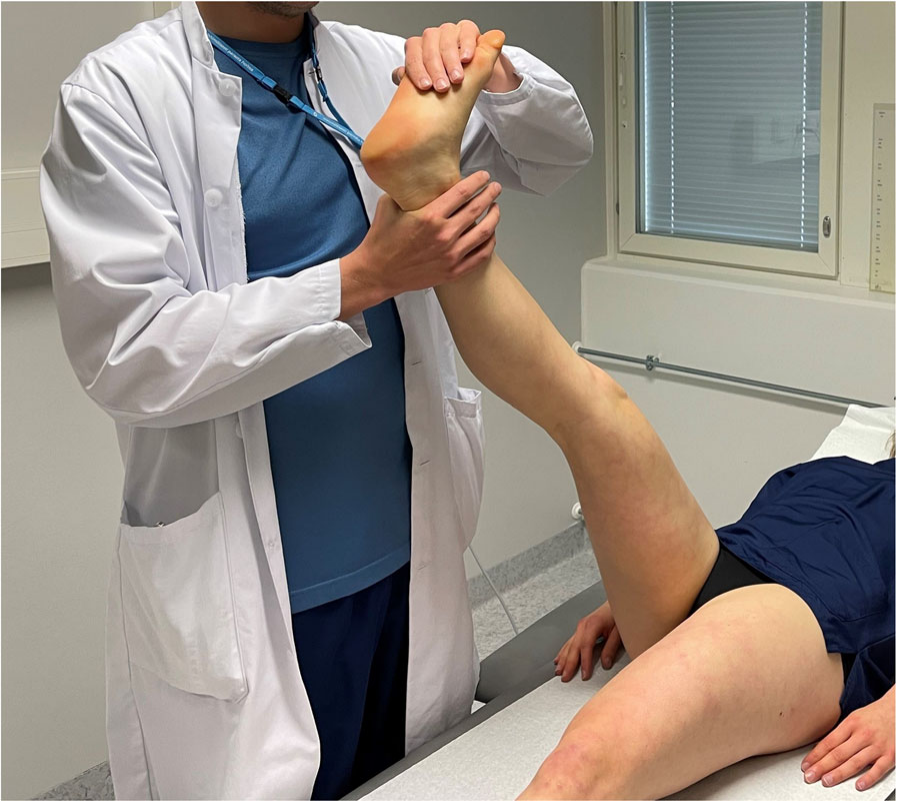
Distal structural differentiation for proximal symptoms with ankle dorsiflexion (also known as Bragard test)

**Fig. 2:**
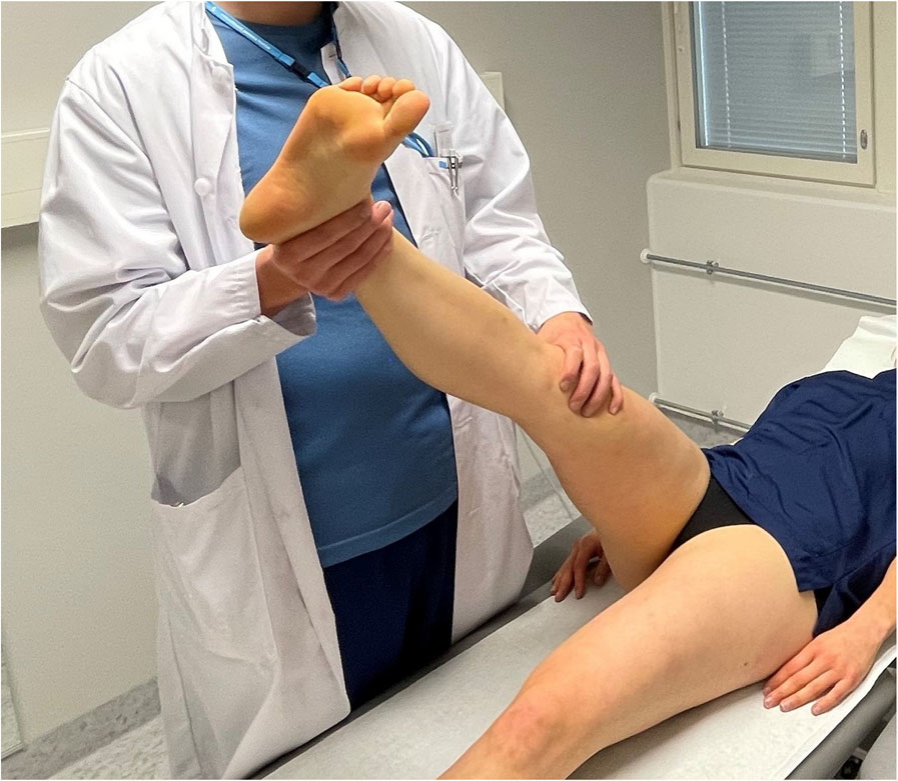
Proximal structural differentiation for distal symptoms with hip internal rotation

For subjects whose symptoms occurred in the gluteal and/or hamstring areas, the differentiating movement was passive ankle dorsiflexion (i.e. distal differentiation). This was executed by moving the examiner’s proximal hand from above the knee to the ball and toes of the foot while keeping the SLR angle constant and dorsiflexing the ankle gently from neutral (loose) position to 90° of dorsiflexion (as in Bragard test, Fig. 1). Ankle dorsiflexion applies tension to, and moves, the sciatic nerve distally without moving biceps femoris muscle [28, 29]. For the proximal nerve movement for patients with distal reproduction of symptoms (below the knee), hip internal rotation was used to differentiate the evoked responses to be of neural origin [30]. This was performed with the same hand positioning as described earlier with the SLR by turning the examiner’s wrists to produce internal rotation to the hip joint while keeping the SLR angle at evoked responses stable and avoiding adduction of the hip (Fig. 2). In case the SLR did not provoke any responses before or at 90° of hip flexion, the test was judged negative and no structural differentiation was performed. If the subject’s symptoms evoked by the test increased by structural differentiation, the ESLR was ruled to contain a neural aspect, and deemed ‘positive’. Conversely, the test was deemed negative if the structural differentiation did not increase the SLR-provoked symptoms.

Two aspects were required for a positive test: i) reproduction of the subject’s clinical symptoms during the SLR, and ii) increase of those symptoms with differentiating movement (hip rotation or dorsiflexion). An important remark with the ESLR is that it is imperative to perform the differentiating movement only at a location that is anatomically different from the location of the evoked symptoms, i.e. proximal symptoms ➔ distal differentiation, and vice versa. In case the differentiating movement was performed on the same anatomical location as the evoked symptoms, it will likely cause some symptoms/sensations on the site of provoked symptoms, which can be confused with as the worsening of sciatic symptoms.

### Statistical analysis

The data were analyzed using Microsoft Excel and IBM SPSS Statistics, version 26. Positive/negative findings of Study Controller and both Examiners 1 and 2 were compared and interrater agreement percentage was calculated. Observed Agreement (P_0_) and prevalence of the index condition (P_index_) were calculated in order to better compute and interpret resulting Kappa values. The sample size of 40 was required for the Kappa statistic to be significantly greater than 0.40 (assuming 80% power and 0.05 level of significance) [25, 31], where 0.40 represents the value of the null hypothesis. Positive/negative findings of both Examiners 1 and 2 were cross-tabulated and the Cohen’s Kappa statistic was used for interrater reliability between the examiners for the ESLR result. *P*-values and 95% confidence intervals (95%CI) were calculated. The ESLR results were also compared with the traditional SLR results and interrater agreement (overall agreement percentage and Kappa) were calculated between the Examiners’ ESLR result and the traditional SLR.

## Results

The study group consisted of 40 subjects, 25 women and 15 men: mean age was 41 years (range 22–64 years), height 170 ± 9 cm (mean ± standard deviation), and weight 80 ± 23 kg. The mean ESLR angle for the sciatic group was 60 ± 19° (range 30°- 85°) while control group’s mean ESLR angle was 84° ± 8° (range 70°- 90°).

The Overall Agreement or Observed Agreement P_0_ based on a 2 × 2 contingency table and the data in Table 2 was calculated with the following formula

$$ \mathrm{Po}=\frac{a+d}{n}=\frac{18+19}{40} = \mathrm{0,925} $$

**Table 2:**
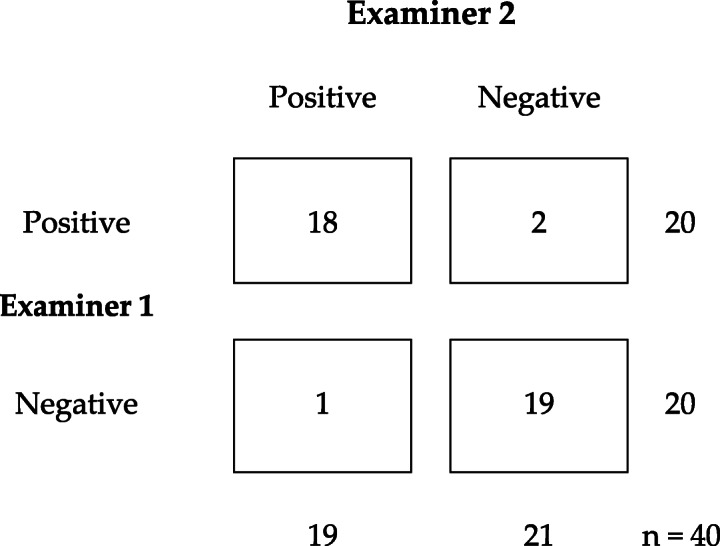
Crosstabulation for ESLR result between Examiners 1 and 2

In reproducibility studies, the prevalence of the index condition P_index_ is synonymous with the frequency of all positive judged diagnostic procedures (the index condition) by the observers. Per a 2 × 2 contingency table and the data in Table 2 the P_index_ was equal to:

$$ \mathrm{Pindex}=\frac{\left(a+\frac{b+c}{2}\right)}{n}=\frac{18+1,5}{40} = 0,4875 $$

The interrater agreement between Examiner 1 and Examiner 2 for the ESLR was 0.85 (*p* < 0.001, 95%CI: 0.71–0.99) translating to almost perfect agreement as measured by Cohen’s Kappa (Table 2). The overall agreement rate between Examiners 1 and 2 was 92.5, and 95.0% between E1/SC, and again 97.5% between E2/SC. There were 3/40 subjects whose SLR result was not unanimous: 2 in the symptomatic group (ESLR+ 80° with both subjects) and one in the control group (hamstring tightness at 70°).

When the ESLR was compared to the traditional SLR, we found that the agreement was only partial: All 20/20 subjects in the control group (ESLR-) we also judged as ‘negative’ with the traditional SLR. In the sciatic (ESLR+) group 10/20 were determined ‘positive’ with the traditional SLR. However, 6/20 traditional SLRs in the sciatic group were determined negative due to the hip flexion angle reaching over 70 degrees, and 4/20 were negative as the evoked symptoms with the traditional SLR were limited to hamstring/gluteal region. The overall agreement rate between the ESLR and traditional SLR results was 75% (30/40). Kappa values between the traditional SLR and the E1’s or E2’s ESLR results were 0.50 (*p* < 0.0001; 95%CI 0.27–0.73) and 0.54 (p < 0.0001; 95%CI 0.30–0.77), respectively. The crosstabulations are presented in Tables 3 and 4. General agreement between the ESLR and traditional SLR was 0.50 (p < 0.0001; 95%CI 0.27–0.73).

**Table 3:**
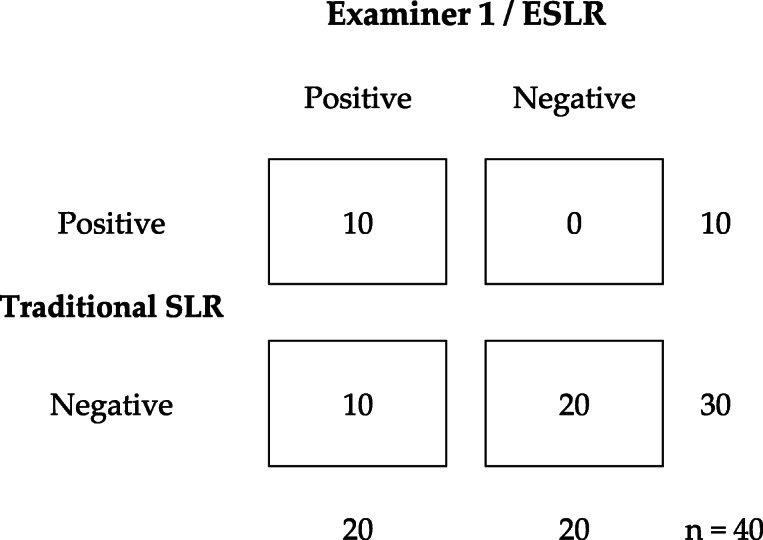
Crosstabulation between Examiners 1’s ESLR and traditional SLR’s result

**Table 4:**
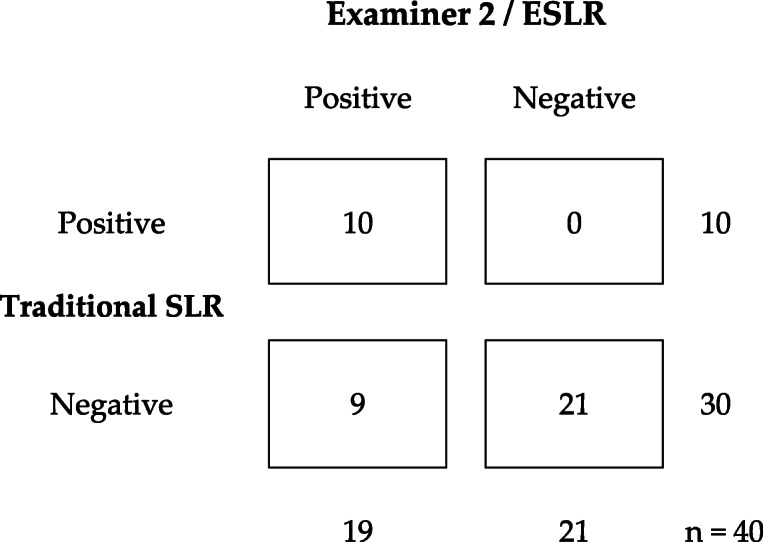
Crosstabulation between Examiners 2’s ESLR and traditional SLR’s results

## Discussion

For the ESLR definition and location-specific structural differentiation movements (ankle dorsiflexion and hip internal rotation), we showed excellent reliability because there was almost perfect agreement between i) the blinded examiners and ii) the examiners and study controller. When compared to the traditional SLR results the interrater agreement was only moderate.

Criticism of the SLR has been about its heterogeneity in diagnosing lumbar disc hernia**,** particularly specificity [10, 11]. This is likely due to an imperfect concept as to what the test measures. Many mechanisms and pathologies can relate to radicular pain and the SLR. Even though mechanical compression (disc hernia) can; mechanosensitivity, inflammatory issues, and impairment of neural movement, can also be important factors [3, 4, 7–9]; and lumbar disc hernias are often asymptomatic [14]. The SLR is indirect because it tests physical mechanisms such as mechanical function (excursion) and sensitivity, not pathology, disease, or anatomical changes, as noted also by Walsh and Hall [32].

We extended the SLR by adding differentiation movements to it based on the meticulous scientific data on the effects of different components of the SLR to the nervous system [16, 20–24, 28]. Moreover, as the SLR is employed more than other tests in clinical practice worldwide with LBP, implementing on the execution and interpretation of this test may create a more relevant impact in the scientific and clinical community. By adding a differentiating maneuver to the SLR, a test capable of emphasizing neural symptoms over musculoskeletal is created. These modifications were selected so the examiner can move the nerves without moving the musculoskeletal structures at the site where the symptoms were provoked [28–30]. Specifically, if there is mechanosensitivity or tension in the neural structures, neural movement generated from asymptomatic musculoskeletal location causes the symptom aggravation by which it can be separated from musculoskeletal symptoms. This is significant, for example, when assessing a patient with a proximal (above the knee) reproduction of symptoms with the SLR: Traditionally the SLR would have been determined as negative, but with the structural differentiation (i.e., the ESLR) it now may be possible to test if the symptoms are evoked from neural tissues. Furthermore, it is possible discern the evoked symptoms easily regardless of at which hip flexion angle they are provoked and hence can discard the angle restrictions suggested with the traditional SLR. The near-perfect interrater agreement for the ESLR not only increases the value of this test, but also reliability and repeatability in interpretation are of paramount importance and represents the central part of this investigation. Also, the moderate agreement found between ESLR and traditionally performed SLR does indicate the potential of ESLR in integrative interpretation as to clear ambiguousness found in traditional SLR testing, especially in situations in which traditional SLR is eliciting symptoms over 70 degrees and when reproduction of symptoms does not occur below the knee.

Reflecting the present study, the lack of a legitimate reference standard (on which to compare the SLR results) can be considered as a weakness. On the other hand, it has been shown that, by using a combination of patient history and clinical findings, sciatic patients can reliably be extracted from the patient population [26, 27]. Accordingly, we used this as a reference standard for the SC to classify and allocate the subjects to sciatic and control groups. Then again, this classification does not play an integral role in interrater reliability/repeatability analysis as it is ultimately a question for the agreement of the ESLR result (positive/negative). Another limitation for the study can be that the traditional SLR was performed by a treating physician and not by a blinded examiner. Our subject sample reflects a realistic patient-care setting in a specialized spine clinic on which the groups have been matched to 20 subjects each, but at the same time it does not represent a realistic population-wide distribution of sciatic and nonspecific low back pain patients. As the proportion of sciatic patients in normal population is significantly lower, this can be considered as a limitation of the study.

This study was designed to test ESLR’s repeatability and interrater agreement on the test result rather than testing how different variables predict the existence of a certain (pathologic) condition. It is noteworthy, that we were able to modify the SLR so that both clinical application and interpretation of the ESLR were reliable and repeatable, and produced constant results between blinded examiners even without the knowledge of patient history, imaging or other clinical tests. The addition of location-based differentiation movements (hip internal rotation or ankle dorsiflexion) to the SLR produces a promising test with which it may be capable to discern neural symptoms from musculoskeletal. This knowledge can lead to a better recognition of patients with sciatic/neural ailments and in planning more sophisticated and focused treatment protocols. In the future, the ESLR needs to be further assessed for its validity and diagnostic performance.

## Conclusions

The extended SLR adds hip internal rotation or ankle dorsiflexion to apply more tension to the neural tissues than the SLR. The ESLR produces constant results in patients with LBP with or without sciatica, and may improve diagnostic ability for detection of a likely neural element. Further studies are still needed for its validity assessment.

## Data Availability

The datasets used and/or analysed during the current study are available from the corresponding author on reasonable request.

## Abbreviations

95%CI: 95% confidence interval
ESLR: Extended straight leg raise test
LBP: Low back pain
MRI: Magnetic resonance imaging
P_0_: Overall Agreement or Observed Agreement
P_index_: Prevalence of the index condition
SLR: Straight leg raise test
